# Effects of verbal instructions and physical threat removal prior to extinction training on the return of conditioned fear

**DOI:** 10.1038/s41598-020-57934-7

**Published:** 2020-01-27

**Authors:** Julia Wendt, Miriam C. Hufenbach, Jörg König, Alfons O. Hamm

**Affiliations:** 1 Department of Psychology, University of Greifswald, Greifswald, Germany; 2 Department of Psychology, University of Potsdam, Potsdam, Germany

**Keywords:** Fear conditioning, Experimental models of disease

## Abstract

Instructions given prior to extinction training facilitate the extinction of conditioned skin conductance (SCRs) and fear-potentiated startle responses (FPSs) and serve as laboratory models for cognitive interventions implemented in exposure-based treatments of pathological anxiety. Here, we investigated how instructions given prior to extinction training, with or without the additional removal of the electrode used to deliver the unconditioned stimulus (US), affect the return of fear assessed 24 hours later. We replicated previous instruction effects on extinction and added that the additional removal of the US electrode slightly enhanced facilitating effects on the extinction of conditioned FPSs. In contrast, extinction instructions hardly affected the return of conditioned fear responses. These findings suggest that instruction effects observed during extinction training do not extent to tests of return of fear 24 hours later which serve as laboratory models of relapse and improvement stability of exposure-based treatments.

## Introduction

Exposure-based treatments of pathological anxiety, which involve confronting patients with their feared stimulus or situation until the fear response declines^1^, are considered as highly efficient^2^. However, about one third of patients undergoing exposure-based treatments may not experience a clinically relevant decline in their pathological anxiety^3^ and another third may experience a relapse, i.e. a return of their pathological anxiety, within 10 years^4^. These findings highlight the relevance of determining factors that moderate both short-term and long-term outcomes of exposure-based treatments. Pavlovian fear conditioning paradigms allow for the investigation of the development, treatment and maintenance of fear under laboratory conditions and typically comprise several experimental phases^5^: During fear acquisition training, a previously unrelated stimulus (conditioned stimulus, CS) is presented together with an aversive event (unconditioned stimulus, US), acquires signal value for the US and eventually elicits a conditioned fear response (CR). During extinction training, the CS is presented without the US and the CR gradually declines which is why extinction procedures serve as a laboratory analogue for exposure-based treatments^6^. Subsequent retention tests which entail the re-exposure to the CS after experimental manipulations and/or a period of time are used to investigate the maintenance of the extinction memory trace. Thus, retention tests are used to model long-term stability of fear reduction and relapse after exposure-based treatments^7^.

Verbal instructions about CS-US associations influence conditioned responding when given prior to acquisition training^8^ as well as when given prior to extinction training^9^. Instructions given prior to extinction training serve as a laboratory analogue for cognitive interventions which often accompany exposure treatments, e.g., in form of guided threat reappraisal^10^. In general, instructions informing participants about the subsequent non-occurrence of the US seem to facilitate extinction learning across different outcome measures^9^. So far, however, research on this topic mostly focused on conditioned skin conductance responding (SCR) and found that instructions often induce an immediate extinction which can be observed even in the first trial of extinction training^11–13^. Facilitating effects of instructions have been observed for the extinction of conditioned fear-potentiated startle responses (FPS) as well^11,14–16^, whereas conditioned CS valence ratings seem to be somewhat immune against instructions^13,15^. In addition, the removal of the physical threat (e.g., by removing the electrode used to deliver an electrotactile US) does not seem to moderate the influence of verbal instructions on extinction learning as indexed by SCRs and conditioned valence ratings^13^, but its effects on the extinction of conditioned FPSs as well as on extinction retention have never been systematically investigated.

Differences between outcome measures have been attributed to the multiple processes involved in fear learning, i.e., cognitive relational^17^ or signal^18^ learning as indexed by conditioned SCRs^19^ and US expectancy ratings^20^, affective learning as indexed by conditioned FPSs^21^, and evaluative conditioning as indexed by conditioned CS liking or valence ratings^22^. According to these assumptions, instructions given prior to extinction may immediately establish a CS-noUS association as expressed by indicators of relational learning, whereas affective learning is facilitated but additional congruent experiences may be needed to abolish defensive responding. The physical threat removal helps to establish a predictable, safe context^23^ and, thus, may increase the effects of verbal instructions on affective learning during extinction training as indexed by FPSs. Finally, the liking of the CS which signaled the US in the past (i.e., evaluative conditioning) is hardly affected by verbal instructions unless specifically targeted with positive information about the CS^24^.

In contrast to these short-term effects, research on the effects of verbal instructions given prior to extinction training on the return of fear is sparse which limits the potential clinical relevance of these findings. Two studies used between-group designs (instruction vs. no instruction) to investigate the effects of either contingency instructions prior to extinction training^11^ or instructions targeting CS valence^24^ on reinstatement of conditioned fear and found no reinstatement of differential SCRs in either group, but reinstatement of conditioned FPS in both groups independent of instructions^11^. Another study^25^ found increased extinction retention only for a group in which safety information was given not only prior to extinction training on day 1, but also repeated prior to the retention test on day 2. Here, however, conditioned responding was measured only with SCRs, although research on the effects of instructions on extinction learning cited above suggests differential effects depending on the outcome measure. In addition, physical threat removal prior to extinction training may moderate subsequent extinction retention depending on whether the threat is present during the retention test or not.

**Figure 1:**
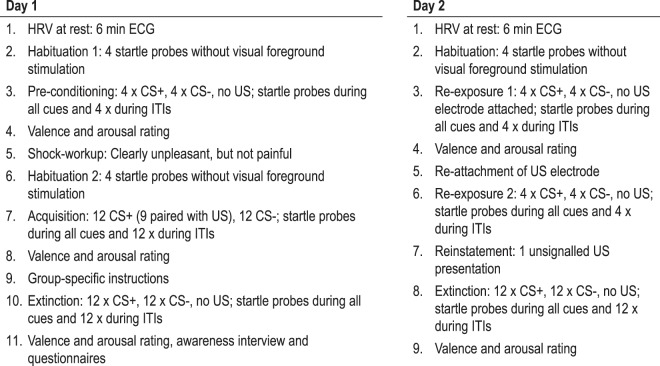
Procedural details on experimental phases, startle noise presentations and rating points over both days of measurement; ECG = electrocardiogram; CS = conditioned stimuli; US = unconditioned stimulus; ITI = inter-trial interval.

Thus, we conducted a differential cue conditioning study in which acquisition and extinction training took place on day 1 whereat one group received no instructions prior to the extinction training (uninstructed extinction, UE group), another group was informed that no further US will be applied during subsequent trials (verbally instructed extinction, VEI group) and in a third group we removed the physical threat in addition to the verbal instruction (verbally instructed extinction plus US electrode removal, VIE+ R group). On day 2, we tested spontaneous recovery of the CR by re-exposing all groups to the CSs first in the absence of the physical threat, i.e., without the US electrode attached, then again in presence of the physical threat, i.e., with the US electrode attached. Then an unsignaled US was presented to test the effects of extinction instructions on reinstatement of fear. Skin conductance and fear-potentiated startle responses were measured during all experimental phases and CS valence ratings were obtained after each of the experimental phases (see Fig. 1 for procedural details on experimental phases, startle noise presentations and ratings). Based on previous findings and theoretical considerations outlined above, we expected an immediate extinction of conditioned SCRs in both instructed groups as well as a facilitated extinction of conditioned FPSs with an earlier onset in the VIE+ R group, but no effect of instructions on conditioned CS valence ratings. Assuming that the physical threat removal during extinction training may provide contextual information that influence extinction retention, we expected increased extinction retention in the VIE+ R group when tested without the US electrode, but decreased extinction retention in presence of the physical threat, i.e., with the US electrode attached. Due to the lack of previous research on this topic and because contextual features seem to influence return of fear across different learning processes^7^, we had no specific hypotheses for different outcome measures.

## Results

### Startle blink responses

#### Day 1: Fear acquisition and extinction

A linear mixed model testing the fixed effects trial type (CS+ vs. CS- vs. ITI) and instruction group (VIE vs. VIE+ R vs. UE) during the pre-conditioning phase revealed that instruction group (*F*(2, 333.5) = 6.674, *p* = 0.001, *partial R²* = 0.080) but not trial type (*F* < 1, *p* = 0.393) significantly affected startle magnitude. Sidak-corrected pairwise comparisons confirmed that the UE group showed generally smaller startle magnitudes than the VIE (*mean difference* = −1.50, *p* = 0.067, 95% CI [−3.08, 0.07]) and the VIE-R group (*mean difference* = −2.37, *p* = 0.001, 95% CI [−3.96, −0.79]) during pre-conditioning. Testing the raw scores during the pre-conditioning phase confirmed these findings, i.e., significant main effect of instruction group (*F*(2, 156.8) = 6.303, *p* = 0.002, *partial R²* = 0.161) and Sidak-corrected pairwise comparisons (VIE vs. UE group: *mean difference* = −14.63, *p* = 0.075, 95% CI [−30.28, 1.03]; VIE+ R vs. UE group: *mean difference* = −22.75, *p* = 0.002, 95% CI [−38.52, −6.98]). Thus, we took the average responding during pre-conditioning into account as a covariate for all subsequent tests on startle magnitudes during acquisition and extinction training.

**Figure 2:**
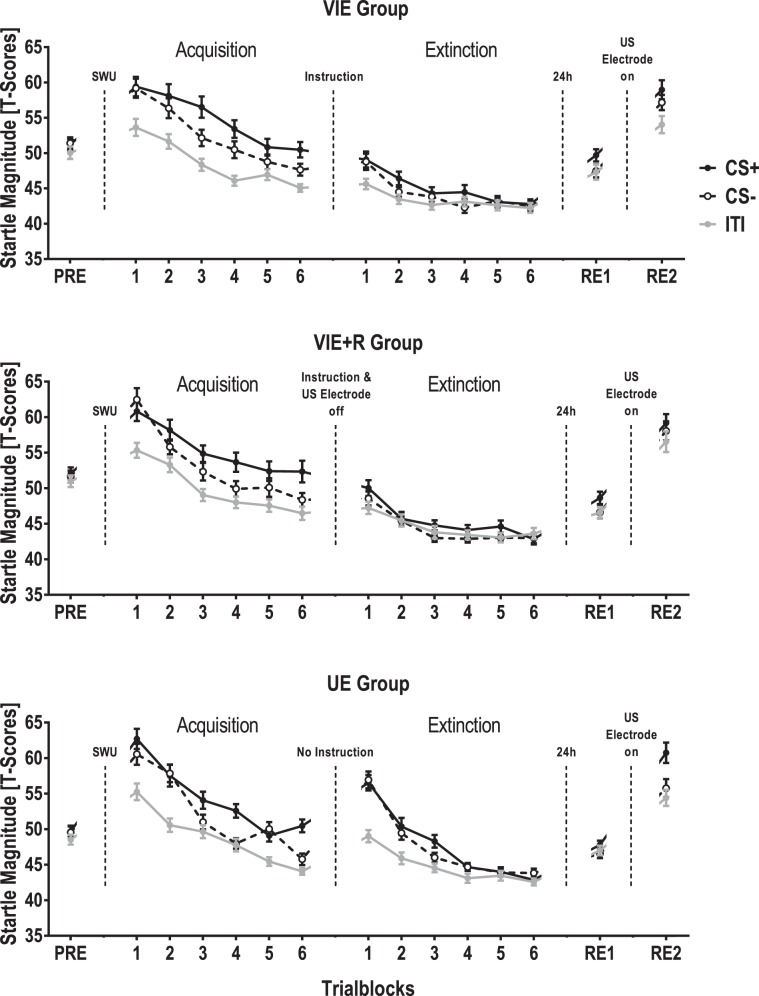
Startle blink magnitudes [T-scores] presented separately for participants who received verbal instructions that no further US will be applied prior to extinction training (VIE group, upper panel), participants for whom the US electrode was removed in addition to verbal instructions (VIE-R group, middle panel) and participants who received no instruction prior to extinction training (UE group, lower panel). For the purpose of visualization, responses during CS +, CS- and ITIs are averaged for the four trials of pre-conditioning (PRE), for each two trials during acquisition and extinction training and for the each four trials of the first re-exposure to the CSs after 24 h (RE1) and the second re-exposure to the CSs after reattachment of the US electrode (RE2). Error bars represent standard errors of the mean.

A linear mixed model testing the fixed effects trial type and instruction group as well as the fixed effect trial number (to account for learning effects) during acquisition training revealed that startle magnitudes were significantly affected by trial type (*F*(2, 1153.1) = 103.551, *p* < 0.001, *partial R²* = 0.359), trial number (*F*(11, 2221.5) = 57.533, *p* < 0.001, *partial R²* = 0.570) and their interaction (*F*(22, 2213.0) = 2.360, *p* < 0.001, *partial R²* = 0.047), but not by the instruction group (*F*(2, 1048.3) = 1.176, *p* = 0.309) when controlling for the average responding during pre-conditioning and its association with the experimental groups. Follow-up linear mixed models testing either CS+/CS- differentiation or CS+/ITI potentiation confirmed that acquisition training led to an differentiation between CS+ and CS− (*F*(1, 752.6) = 25.864, *p* < 0.001, *partial R²* = 0.069) which progressively increased (CS differentiation x trial number: *F*(11, 1478.8) = 2.618, *p* = 0.003, *partial R²* = 0.039) as well as a pronounced potentiation of startle responses elicited during CS+ in comparison with those elicited during the ITI (CS+ potentiation: *F*(1, 767.3) = 210.172, *p* < 0.001, *partial R²* = 0.548) that developed during acquisition training (CS potentiation x trial number: *F*(11, 1466.1) = 2.127, *p* = 0.016, *partial R²* = 0.032); see Fig. 2.

A linear mixed model testing the fixed effects trial type and instruction group as well as the fixed effect trial number (to account for learning effects) during extinction training revealed that startle magnitudes were significantly affected by trial type (*F*(2, 1071.0) = 22.737, *p* < 0.001, *partial R²* = 0.085), trial number (*F*(11, 2164.2) = 57.343, *p* < 0.001, *partial R²* = 0.583) and their interaction (*F*(22, 2153.2) = 2.800, *p* < 0.001, *partial R²* = 0.100). In addition, instruction groups differed both in their general response magnitudes during extinction training (*F*(2, 957.1) = 19.455, *p* < 0.001, *partial R²* = 0.081) as well as dependent on trial type (*F*(4, 1071.0) = 4.835, *p* < 0.001, *partial R²* = 0.036) and trial number (*F*(22, 2165.0) = 4.923, *p* < 0.001,, *partial R²* = 0.100) when controlling for the average responding during pre-conditioning and its association with the experimental groups. Sidak-corrected pairwise comparisons revealed that the UE group showed generally higher startle magnitudes than the VIE (*mean difference* = 2.43, *p* < 0.001, 95% CI [1.72, 3.14]) and the VIE-R group (*mean difference* = 2.21, *p* < 0.001, 95% CI [1.49, 2.93]) during extinction training.

Following up on significant interactions between instruction group and trial type respectively trial number, we computed two further linear mixed models per instruction group, one testing for the effect of trial type and the other testing for the effect of trial number. The first set of models revealed a medium effect of trial type in the UE group (*F*(2, 77.0) = 14.576, *p* < 0.001, *partial R²* = 0.117) driven by pronounced startle potentiation during both CS+ and CS− presentations compared to ITIs (Sidak-corrected pairwise comparisons; CS+ vs ITI: *mean difference* = 2.17, *p* = 0.003, 95% CI [0.62, 3.71]; CS- vs ITI: *mean difference* = 2.87, *p* < 0.001, 95% CI [1.36, 4.38]), a small effect in the VIE group (*F*(2, 367.6) = 3.427, *p* = 0.034, *partial R²* = 0.037) driven by startle potentiation during CS+ presentations compared to ITIs (Sidak-corrected pairwise comparisons; CS+ vs ITI: *mean difference* = 1.45, *p* = 0.029, 95% CI [0.11, 2.79]) and a non-significant effect in the VIE-R group (*F*(2, 67.7) = 2.947, *p* = 0.059); see Fig. 2. A Bayesian one-way univariate analyses of variance (ANOVA) with the repeated measure factor trial type revealed a Bayes-factor of 0.23 for the VIE-R group, i.e., supported the hypothesis that startle responses did not differ between trial types in the VIE-R group.

The second set of follow-up models revealed significant effects of trial number in all three instruction groups with the largest effect in the UE group (*F*(1, 809.8) = 44.003, *p* < 0.001, *partial R²* = 1.195), followed by the VIE-R (*F*(11, 707.1) = 11.714, *p* < 0.001, *partial R²* = 0.364) and the VIE group (*F*(11, 707.7) = 8.945, *p* < 0.001, *partial R²* = 0.278). Sidak-corrected pairwise comparisons using the last trial of extinction training as reference confirmed different response reduction time courses in the different groups, that is in the UE group response magnitudes up to the 5^th^ trial were significantly different from the 12^th^ trial (5^th^ vs 12^th^: *mean difference* = 4.30, *p* < 0.001, 95% CI [1.91, 6.70]; 6^th^ vs 12^th^: *mean difference* = 2.33, *p* = 0.068, 95% CI [−0.88, 4.75]), whereas in the VIE group significant differences compared to the 12^th^ trial were observed up to the 3^rd^ trial (3^rd^ vs 12^th^: *mean difference* = 2.68, *p* = 0.017, 95% CI [0.28, 5.08]; 4^th^ vs 12^th^: *mean difference* = 1.61, *p* = 0.482, 95% CI [−0.80, 4.02]) and in the VIE-R group up to the 2^nd^ trial of extinction training (2^nd^ vs 12^th^: *mean difference* = 3.26, *p* = 0.001, 95% CI [0.95, 5.57]; 3^rd^ vs 12^th^: *mean difference* = 1.99, *p* = 0.165, 95% CI [−0.36, 4.33]); see Fig. 2.

Additionally, we investigated immediate extinction effects by testing the fixed effects trial type and instruction group for the first extinction trial only and found significant main effects of trial type (*F*(2, 207.5) = 18.490, *p* < 0.001, *partial R²* = 0.356), instruction group (*F*(2, 120.5) = 5.197, *p* = 0.007, *partial R²* = 0.173) as well as their interaction (*F*(4, 207.5) = 3.269, *p* = 0.013, *partial R²* = 0.126). Follow-up linear mixed models conducted within instruction groups revealed a large effect of trial type in the first trial of extinction training in the UE group (*F*(2, 77.0) = 14.576, *p* < 0.001, *partial R²* = 0.757) driven by pronounced startle potentiation during both CS and CS- presentations compared to ITIs (Sidak-corrected pairwise comparisons; CS+ vs ITI: *mean difference* = 7.77, *p* = 0.001, 95% CI [2.63, 12.90]; CS- vs ITI: *mean difference* = 10.44, *p* < 0.001, 95% CI [5.66, 15.22]), a significant effect in the VIE group (*F*(2, 60.8) = 4.839, *p* = 0.011, *partial R²* = 0.318) again driven by potentiation during both CS+ and CS− presentations compared to ITIs (Sidak-corrected pairwise comparisons; CS+ vs ITI: *mean difference* = 4.61, *p* = 0.020, 95% CI [0.56, 8.66]; CS- vs ITI: *mean difference* = 3.85, *p* = 0.021, 95% CI [0.46, 7.24]) and a non-significant effect in the VIE-R group (*F*(2, 67.7) = 2.947, *p* = 0.059). However, a Bayes-factor of 0.57 for the VIE-R group offers only limited support for the null hypothesis.

Critically, during the last two trials of extinction training neither trial type nor instruction group or their interaction significantly affected startle magnitude (all *F*s < 1, all *p*s ≥ 0.443). Bayesian one-way ANOVAs with the repeated measure trial type revealed Bayes factors of 0.09 (UE), 0.02 (VIE) and 0.03 (VIE+ R) which confirmed a complete extinction of conditioned fear-potentiated startle responses in all experimental groups as a baseline for return of fear tests on day 2.

#### Day 2: Return of fear

Linear mixed models testing the fixed effects trial type, reattachment and instruction group on startle magnitudes during re-exposure to the CSs 24 hours after extinction training were affected by trial type (*F*(2, 1312.6) = 12.422, *p* < 0.001, *partial R²* = 0.032) and, most strongly, by the reattachment of the US electrode (*F*(1, 715.2) = 405.256, *p* < 0.001, *partial R²* = 1.133). Sidak-corrected pairwise comparisons revealed that startle magnitudes elicited during re-exposure to the CS+ were higher than those elicited during CS- presentations (CS differentiation: *mean difference* = 2.09, *p* = 0.001, 95% CI [0.76, 3.42) and ITIs (CS+ potentiation: *mean difference* = 2.68, *p* < 0.001, 95% CI [1.32, 4.03). We also observed a small main effect of instruction group (*F*(2, 576.1) = 3.819, *p* = 0.022, *partial R²* = 0.027), however, Sidak-corrected pairwise comparisons failed to confirm significant differences between groups. We did not observe any significant interactions between instruction group, trials type and reattachment of the US electrode (all *F*s ≤ 1.438, all *p*s ≥ 0.238).

Although already suggested by the non-significant interaction between trial type and reattachment of the US electrode, we specifically tested return of fear during the first re-exposure to the CSs and found a small effect of trial type (*F*(2, 632.3) = 3.954, *p* = 0.020, *partial R²* = 0.025) even though the US electrode was not even reattached. Sidak-corrected pairwise comparisons revealed that the effect of trial type during the first re-exposure to the CSs was mainly driven by increased startle responses during CS+ compared to CS- trials (CS differentiation: *mean difference* = 1.52, *p* = 0.022, 95% CI [0.16, 2.87]) and to a lesser degree by a potentiation of CS+ responses compared to ITIs (CS+ potentiation: *mean difference* = 1.26, *p* = 0.099, 95% CI [−0.16, 2.69]); see Fig. 2.

**Figure 3:**
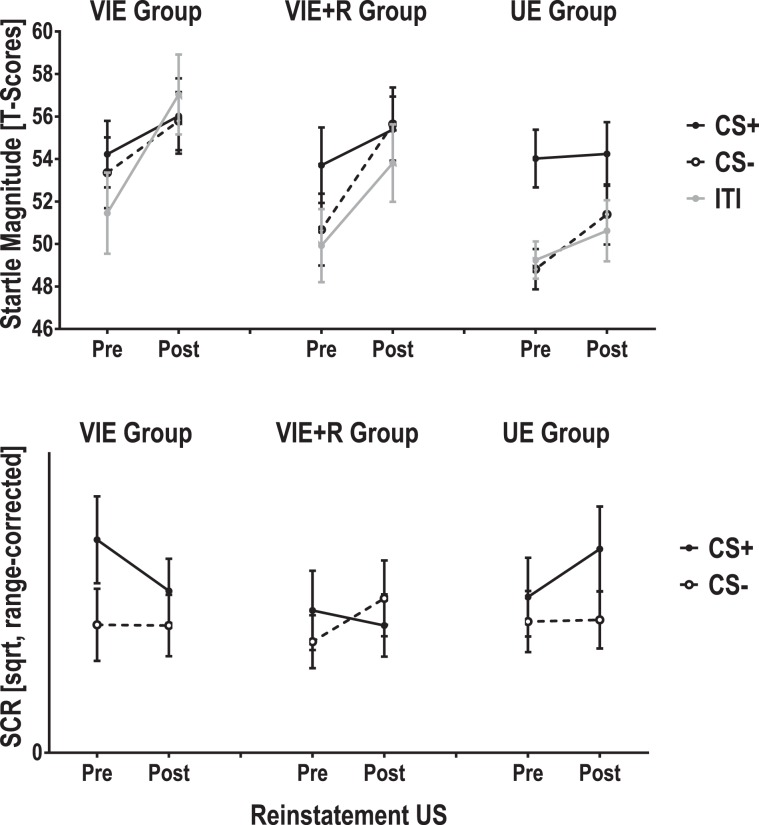
T-transformed startle response magnitudes (upper panel) and square-root-tranformed (sqrt) and range-corrected skin conductance response (SCR) magnitudes (lower panel) for CS trials immediately before (pre) and after (post) an unsignaled US presentation.

Even though the visual inspection of reinstatement patterns indicated differences between experimental groups (Fig. 3, upper panel), the linear mixed model confirmed only significant main effects of trial type (*F*(2, 457.2) = 4.886, *p* = 0.008, *partial R²* = 0.043) and, most pronounced, of reinstatement (*F*(1, 457.4) = 16.228, *p* < 0.001; *partial R²* = 0.070) but no significant interactions between these fixed effects or with the instruction groups (all *F*s < 1.615, all *p*s ≥ 0.200). Sidak-corrected pairwise comparisons revealed that the main effect of trial type was driven by potentiation of startle responses elicited during CS+ compared to CS- trials (CS differentiation: *mean difference* = 1.90, *p* = 0.075, 95% CI [−0.13, 3.93]) as well as between CS+ and ITIs (CS+ potentiation: *mean difference* = 2.53, *p* = 0.009, 95% CI [0.50, 4.56]).

### Skin conductance responses

#### Day 1: Fear acquisition and extinction

**Figure 4:**
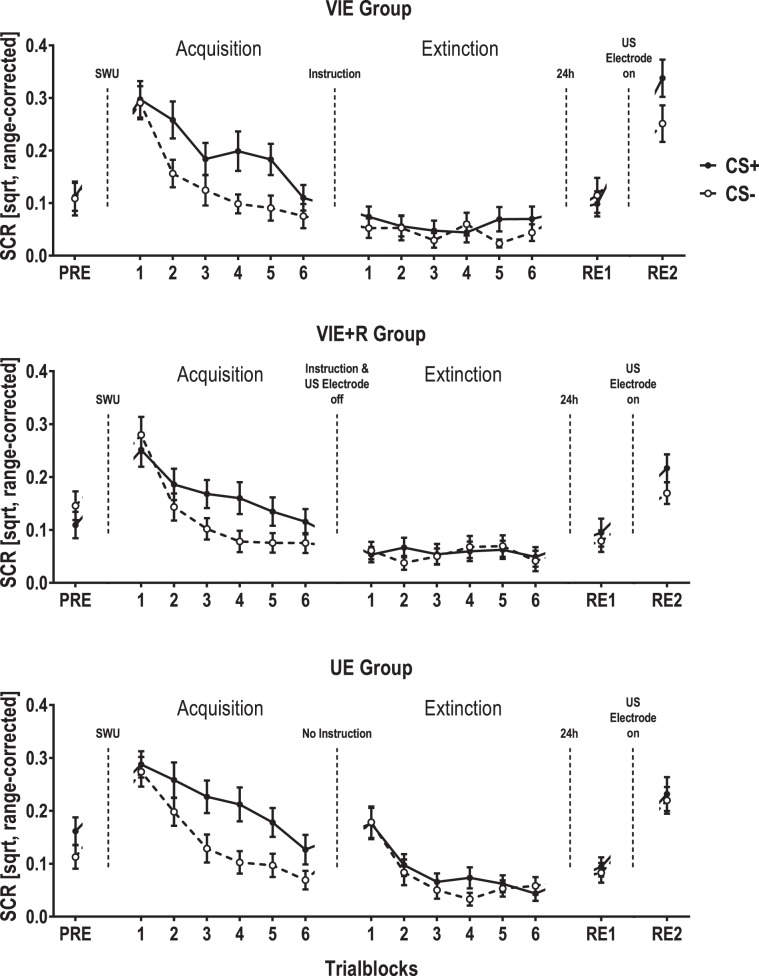
Square-root transformed (sqrt) and range-corrected skin conductance responses (SCRs) [T-scores] presented separately for participants who received verbal instructions that no further US will be applied prior to extinction training (VIE group, upper panel), participants for whom the US electrode was removed in addition to verbal instructions (VIE-R group, middle panel) and participants who received no instruction prior to extinction training (UE group, lower panel). Responses during CS +, CS- and ITIs are averaged for the four trials of pre-conditioning (PRE), for each two trials during acquisition and extinction training and for the each four trials of the first re-exposure to the CSs after 24 h (RE1) and the second re-exposure to the CSs after reattachment of the US electrode (RE2). Error bars represent standard errors of the mean.

A linear mixed model testing the fixed affects CS type (CS+ vs. CS−) and instruction group revealed that during pre-conditioning, neither instruction group nor CS type (both *F*s < 1, *p*s ≥ 0.327) or their interaction (*F*(2, 582.1) = 2.544, *p* = 0.079) significantly affected SCR magnitudes. As evident from Fig. 4 the almost significant interaction between instruction group and CS type resulted from increased SCRs to the CS- in the VIE+ R group and increased SCRs to the CS+ in the UE group. These differential effects were not due to participants’ gender, CS+ face or stimulus order which were all balanced between instruction groups (all Χ²s ≤ 1.27, all *p*s ≥ 0.530). Thus, to control for these baseline differences, we introduced CS differences during pre-conditioning as a covariate in all subsequent tests on SCR magnitudes during acquisition and extinction training.

A linear mixed model testing the fixed effects CS type, trial number and instruction group during acquisition training revealed that SCR magnitudes generally decreased (trial number: *F*(11, 1555.1) = 17.549, *p* < 0.001, *partial R²* = 0.248), but CS+ presentations elicited increased SCR magnitudes compared to CS- presentation (CS type: *F*(1, 601.9) = 31.291, *p* < 0.001, *partial R²* = 0.104) and this differentiation developed with increasing training trials (CS type x trial number: *F*(11, 1526.7) = 2.170, *p* = 0.014, *partial R²* = 0.031). SCRs during acquisition training were not significantly influenced by the instruction group (*F*(2, 517.1) = 2.723, *p* = 0.067) nor, more importantly, by any interaction between groups and other fixed effects (all *F*s ≤ 1, all *p*s ≥ 0.527) when controlling for differential CS responding during pre-conditioning and its association with the experimental groups.

A linear mixed model testing the fixed effects CS type, trial number and instruction group during extinction training revealed the SCR magnitudes no longer differed between CS+ and CS− presentations (CS type: *F*(1, 683.7) = 1.731, *p* = 0.189), but confirmed a main effect of instruction group (*F*(1, 600.5) = 5.588, *p* < 0.001, *partial R²* = 0.019) as well as an interaction between instruction group and extinction trial number (*F*(1, 1482.0) = 2.345, *p* < 0.001, *partial R²* = 0.003) when controlling for SCR baseline effects. Following up on this interaction with ANOVAs with the fixed effect trial number conducted separately for each instruction groups revealed that SCRs significantly changed during the course of extinction training in the UE group (*F*(11, 862) = 8.465, *p* < 0.001, *partial R²* = 0.216), but not in the instructed groups (VIE and VIE+ R: both *F*s ≤ 0.705, both *p*s ≥ 0.734). Bayesian ANOVAs with the repeated measure trial number revealed Bayes factors of 0.00 for both the VIE and VIE+ R group, thus, conforming the null hypothesis that SCR magnitudes did not differ between extinction trials.

Additionally, we investigated immediate extinction effects by testing the fixed effects CS type and instruction group for the first extinction trial only. Here, we found a large main effect of instruction group (*F*(2, 101) = 8.726, *p* < 0.001, *partial R²* = 0.346), but no main effect of CS type or their interaction (both *F*s ≤ 0.914, both *p*s ≥ 0.404). Sidak-corrected pairwise comparisons revealed that SCRs during the first extinction trial of both CS types were higher in the UE group than in the VIE (*mean difference* = 0.138, *p* = 0.001, 95% CI [0.05, 0.23]) and the VIE+ R group (*mean difference* = 0.133, *p* = 0.001, 95% CI [0.05, 0.22]) with no difference between instructed groups (*mean difference* = 0.01, *p* = 0.999, 95% CI [−0.08, 0.09]). A Bayesian ANOVA revealed a Bayes factor of 0.10, i.e. supporting the hypothesis that SCRs during the first extinction trials did not differ between VIE and VIE+ R groups.

During the last two trials of extinction training neither CS type nor instruction group or their interaction significantly affected SCR magnitude (all *F*s < 1, all *p*s ≥ 0.507). Bayesian one-way ANOVAs with the repeated measure CS type revealed Bayes factors of 0.17 (UE), 0.22 (VIE) and 0.14 (VIE+ R) which confirmed a complete extinction of conditioned SCRs in all experimental groups as a baseline for return of fear tests on day 2.

#### Day 2: Return of fear

During re-exposure to the CSs after 24 hours, SCRs were strongly increased by the reattachment of the US electrode (*F*(1, 1213.3) = 159.285, *p* < 0.001, *partial R²* = 0.263) and varied between instruction groups (*F*(2, 331.4) = 5.124, *p* = 0.006, *partial R²* = 0.062). Separate tests for the two re-exposure phases revealed no effect of CS type during re-exposure without the US electrode (*F* < 1, *p* = 0.736) which was confirmed by a corresponding Bayes factor of 0.09, but a significant increase of SCRs during CS+ compared to CS- presentations after reattachment of the US electrode (*F*(1, 395.1) = 5.884, *p* = 0.016, *partial R²* = 0.030). In addition, the main model revealed a significant interaction between instruction group and reattachment of the US electrode (*F*(1, 1213.2) = 3.667, *p* = 0.026, *partial R²* = 0.006). Follow-up univariate analyses of variance conducted separately for the instruction groups revealed the largest increase of SCRs in the VIE group (*F*(1, 515) = 78,649, *p* < 0.001, *partial R²* = 0.305), followed by the UE (*F*(1, 552) = 55.458, *p* < 0.001, *partial R²* = 0.201) and then the VIE-R group (*F*(1, 522) = 38.632, *p* < 0.001, *partial R²* = 0.148).

After presentation of an unsignaled US, we did not observe any reinstatement effects of conditioned SCRs (main effect of reinstatement and interactions between reinstatement, CS type and instruction group: all *F*s < 1, all *p*s ≥ 0.396; see Fig. 3, lower panel).

### CS valence ratings

#### Day 1: Fear acquisition and extinction

To test for baseline differences in CS valence ratings, we conducted a linear mixed model with the fixed effects of CS type and instructions group on ratings obtained after the pre-conditioning phase. The test confirmed that Cs valence ratings were not significantly influenced by CS type, instruction group or their interaction during before the acquisition training started (all *F*s ≤ 2.335, all *p*s ≥ 0.101).

**Figure 5:**
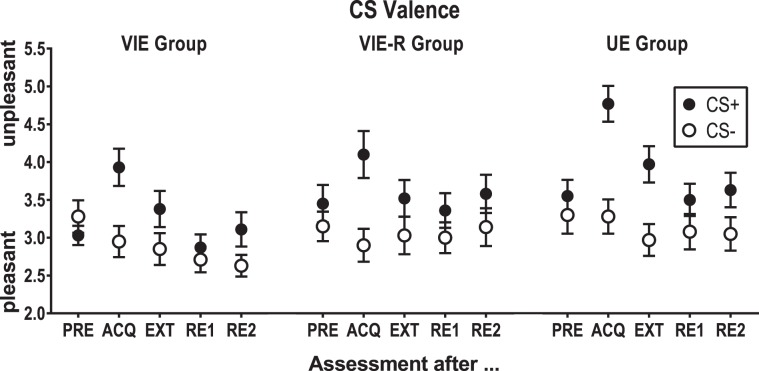
On day 1, CS valence was assessed immediately after pre-conditioning (PRE), acquisition training (ACQ), extinction training (EXT), on day 2 immediately after the first re-exposure to the CSs without the US electrode (RE1) as well as after completion of the experiment, i.e., after the second re-exposure to the CSs with the US electrode attached, exposure to an unsignaled US and subsequent extinction trials (RE1). Group means are depicted along with standard errors.

To test for learning effects on day 1, we conducted a linear mixed model with the fixed effects CS type, time of query (assessment after acquisition training vs. assessment after extinction training) and instruction group. We found significant main effects of CS type (*F*(1, 351) = 94.017, *p* < 0.001, *partial R²* = 0.536; i.e., more negative judgments for the CS+), time of query (*F*(1, 351) = 14.006, *p* < 0.001, *partial R²* = 0.080; i.e., generally less negative judgments after extinction than after acquisition training), as well as their interaction (*F*(1, 351) = 7.878, *p* = 0.005, *partial R²* = 0.045). However, we did not observe any main effect of or interaction with the instruction group (all *F*s ≤ 2.431, all *p*s ≥ 0.089), see Fig. 5.

To ensure a comparable baseline for return of fear tests on day 2 in view of the visible differences between groups in Fig. 5 (EXT), we conducted an additional linear mixed model testing the fixed effects of CS type and instructions group as assessed after extinction training only, i.e., the last CS valence assessment on day 1, which confirmed no significant main effect of or interaction with instruction group (both *F*s ≤ 1.440, both *p*s ≥ 0.241). Using Bayesian tests for independent samples to compare differential valence judgments after extinction training in the UE group with the instructed groups, however, revealed Bayes factors of 2.56 (UE vs. VIE) and 2.33 (UE vs. VIE+ R), thus, provided no support for the hypothesis that valence judgments did not differ between groups after extinction training. Moreover, Bayesian one-way ANOVAs with the repeated measure CS type revealed Bayes factors of 28.72 in the UE group, thus, strong evidence for differential CS valence judgments after extinction training, of 3.58 in the VIE group, thus, moderate evidence for differential judgments, and of 2.99 in the VIE-R group, thus, limited support for differential judgments. To control for possible group differences in differential valence judgments after extinction training, we therefore introduced CS differences after extinction training as a covariate in subsequent tests on CS valence on day 2.

## Discussion

In the present study, we tested the effect of instructions given prior to extinction training that no further US will be applied with or without corresponding removal of the US electrode on extinction learning and return of fear. We found that instructions were associated with an immediate extinction of conditioning SCRs irrespective of US electrode removal and facilitated the extinction of conditioning startle blink responses. During re-exposure to the CSs 24 h later, we observed no effect of instructions on the recovery of conditioned startle blink responses and CS valence ratings. After reattachment of the US electrode, the increase in SCRs differed between instruction groups with the largest effect in verbally instructed participants. The presentation of an unsignaled US resulted in a general increase in startle blink magnitudes, but we observed neither a reinstatement of differential startle or SC responding to subsequent CS trials nor any interaction with instructions groups.

### Effects of instructions on extinction learning

We replicated previous findings that instructions are associated with an immediate extinction of conditioned SCRs^11,12^ whether the US electrode is removed or not^13^. Thus, instructions seem to override cognitive relational learning as indicated by conditioned SCRs^19,21^ by immediately establishing a CS-noUS association. This interpretation is further supported by corresponding earlier findings on the immediate extinction of differential US expectancy ratings by way of instructions^11^. The UE group also showed an immediate loss of differential CS responses but caused by a pronounced increase of CS- responses (see Fig. 4, lower panel), i.e., other than in the instructed groups, SCRs were enhanced at the beginning and decreased during the course of extinction training. Similarly, uninstructed participants showed a pronounced increase in FPSs in response to both the CS+ and the CS- at the beginning of the extinction training (see Fig. 2, lower panel). These conspicuous CS- responses after a short break without further instructions may reflect uncertainty or even the expectation of reversed CS-US contingencies in some participants and should be the subject of future investigations.

For the instructed groups, we observed that the extinction of conditioned FPSs^19,21^ is facilitated by instructions but seems to require additional experiences to attain complete extinction which is also in line with previous reports^11^. We add to the existing literature by showing that the removal of the US electrode may slightly accelerate the effects of verbal instructions on the reduction of conditioned FPSs. Thus, unlike what we and others^13^ have observed for conditioned SCRs, the additional removal of the physical threat seems to moderate the effects of instructions on conditioned FPSs which some consider as indices for affective learning^21^. Affective learning results in the activation of the defensive system, i.e., the neural networks involved in threat processing and their communication with those circuits organizing defensive responding^26^, in response to a CS previously paired with an aversive event^21^. Thus, our findings implicate that the physical removal of the threat, i.e., the provision of an extremely potent safety signal, is associated with a slightly more effective inhibition of the defensive system than mere verbal instructions.

Replicating previous findings, we found that neither verbal instructions in themselves^13,15,27^ nor the additional removal of the US electrode^13^ influenced the extinction of conditioned CS valence ratings. However, as indicated by additionally calculated Bayes factors our data also do not support the null hypothesis that instructed and uninstructed groups show no difference of conditioned CS valence ratings after extinction training. In fact, we observed strong evidence for differential CS valence judgments after extinction training in the UE group, but only moderate evidence for differential judgments in the VIE group and limited support for differential judgments in the VIE-R group. Thus, these findings neither clearly support nor disprove the assumption that CS valence ratings indicate yet another form of learning, i.e. evaluative learning, which seems to be not only generally more resistant to extinction than other types of learning^28^, but also more “resistant to cognitive influence”^9^ than other outcome measures.

### Effects of extinction instructions on return of fear

In the present study, we tested the effects of instructed extinction on return of fear by spontaneous recovery of conditioned responses under two conditions: First, participants were re-exposed to the CSs in a safe environment, i.e., without reattachment of the US electrode. Across groups, we observed a recovery of differential FPSs, but not of conditioned SCRs and a decrease in differential CS valence ratings. According to the retrieval model of extinction and return of fear^29^, extinction may be specific to its temporal context and, thus, spontaneous recovery may reflect the dominance of excitatory CS-US associations formed during acquisition training over inhibitory CS-noUS associations formed during extinction training when tests take place outside of its temporal context. Our findings support the assumption that these associations are stored in parallel in different memory systems, an affective fear memory system indexed by FPS responses and a declarative memory system indexed by SCRs^21^. Thus, when tested in a safe context, excitatory associations formed during acquisition training that are retrieved from the affective memory system seem to prevail over inhibitory associations formed during extinction training, whereas the same is not true for associations retrieved from the declarative memory system. In line with this interpretation, the affective memory system is generally considered to be less dependent on the retrieval context^21^. Furthermore, the lack of extinction instruction effects on spontaneous recovery indicate that inhibitory CS-noUS associations formed during extinction training after instructions are not stronger than those formed during uninstructed extinction training when tested in a safe environment.

Findings from the second re-exposure to the CSs, during which the US electrode was reattached confirm these assumptions. Across groups, we observed a pronounced increase in startle magnitudes indicating a general activation of the defensive system, but differential FPS responses to the CS+ and CS− were not significantly affected by the change from a safe to a threatening context. In contrast, we observed a return of differential SCRs only after the reattachment of the US electrode. Thus, recreating the threatening context significantly affected the retrieval of CS-US associations from the declarative memory system, but not from the affective memory system, again underscoring its relative independence from the retrieval context. Apart from these assumptions about associative mechanisms, the SCR findings also fit well with hypotheses about cognitive processes involved in extinction learning and recall, i.e., causal reasoning which allows for predicting an outcome based on observations without necessarily involving associative processes^30^. That is, without the US electrode, participants predict that no US can be delivered and consequently do not show SCR discrimination before, but only after re-attachment of the US electrode. Interestingly, FPS results do not follow this pattern, instead participants showed differential FPSs before the US electrode was re-attached even though causal reasoning must have led them to the prediction that no US will be delivered. Thus, these findings support the interpretation that FPSs are, at least in part, indicative of non-cognitive processes.

In a recent study, Javanbakht and coworkers^25^ provided contingency information prior to extinction training for one CS+ (“US will not follow CS”) and no information for a second CS+ and found that instructions facilitated extinction learning. If instructions were not repeated, however, they observed no difference between the informed and the uninformed CS+ during extinction recall. Similarly, we observed no significant interaction between instruction group and CS type in our data during the extinction recall test in a threatening context (RE2). Here, only the general increase in SCRs in the threatening context was most pronounced in participants who were instructed about the subsequent absence of US applications prior to extinction training indicating that verbal instructions may actually decrease the strength of declarative CS-noUS associations formed during extinction training^31^. Alternatively, instructions may serve as a cognitive context for extinction learning^25^ on the first day and, thus, the lack of instructions on the second day may impair the recall of context-dependent extinction memories^29^. However, these findings are at odds with a recent report of less return of fear assessed with US-expectancy ratings in participants who were instructed prior to extinction training that the probability of US application would be extremely small in the remainder of the experiment^32^. Future research should therefore attempt to replicate the observed effect on the recovery of differential SCRs under threat before assuming a meaningful association between verbal instructions and extinction memory.

Replicating previous findings^11,24^, we did not observe any effects of instructions given prior to extinction training on the reinstatement of conditioned responses by an unsignaled US presentation. According to the retrieval model^29^, re-exposure to the US evokes a newly formed excitatory association between the US and the context which elicits the retrieval of the latent conditioned CS-US association over the competing inhibitory CS-noUS association. Thus, our results further support the assumption that instructions do not influence the strength of extinction memories.

### Limitations and conclusions

Our findings regarding the reinstatement of fear, however, must be interpreted with caution. The incomplete re-extinction of conditioned fear after reattachment of the US electrode may have prevented both the observation of reinstatement main effects as well as effects of instructions given prior to extinction training^33^. Another limitation of this study concerns the assessment of CS valence only after, but not during the experimental phases. Online ratings allow to capture immediate instruction effects and, thus, are especially preferable in this line of research^9^. At least on day 1, however, ratings after each of the experimental phases should have prevented any interference^34^ or renewal^9^ effects and, thus, ensured the general interpretability of the reported CS valence ratings. Finally, differential interpretations of FPSs and SCRs in the context of fear conditioning research must take into account that these two outcome measures are evoked under systematically different conditions, SCRs by the cue onset and FPSs by an additional probe, temporally much closer to a possibly occuring US. That is, instead of tapping into different memory systems, differential findings for both measures might be due to other factors such as threat imminence^35^ which should be systematically explored in future research.

In conclusion, instructions about the subsequent absence of US stimulation given prior to extinction training substantially influence conditioned responding during extinction training, but hardly have any effect on the return of fear assessed 24 hours later under various conditions. That implies that inhibitory CS-noUS associations may be established faster when extinction training is instructed, but inhibitory extinction memories formed after corresponding instructions are not necessarily stronger in the long term. Since return of fear tests are considered a laboratory analogue to relapse and the stability of improvements after exposure treatment of anxiety disorders^7^, our results correspond with meta-analytic clinical evidence that cognitive behavioral and exposure only treatments for anxiety disorders are equally effective^36^. However, it has been shown that contingency information facilitates extinction recall when provided again directly prior to return of fear tests^25^. Moreover, effects of instructions on fear expression and extinction learning vary with characteristics of the participants^8^. Thus, future research may investigate how these procedural and individual characteristics mediate the effect of instructions on return of fear and may provide a point of reference for individualized treatments.

## Materials and Methods

### Participants

This investigation is part of a larger project, thus, details about the sample have already been provided in Wendt *et al*.^37^. Participants were 126 students of the University of Greifswald between the ages of 18 and 35 who were selected based on a telephone interview in which we checked for the following exclusion criteria: impaired hearing or color vision, excessive smoking habits (>10 cigarettes a day), competitive athletes, self-reported diagnosis of a neurological or mental disorder, regular intake of medication that affects the cardiovascular or central nervous system, neurodermatitis and pregnancy. Participants were randomly assigned to one of the three experimental groups stratified by gender and age. All participants gave written informed consent to the experiment approved of by the ethics committee of the University of Greifswald and performed in accordance with the Declaration of Helsinki.

The experiment was prematurely terminated for six participants on day 1 either due to technical difficulties or at their own request leaving a sample of *N* = 120 with *n* = 40 per experimental group. As targeted, gender distribution (UE group: 19 women; VIE group: 20 women; VIE+ R group: 21 women; Χ²(2) = 0.200, *p* = 0.905) and average age (UE group: 22.6 years ± 3.1 *SD*; VIE group: 23.4 years ± 3.0 *SD*; VIE+ R group: 23.2 years ± 2.7 *SD*; *F*(2,117) = 0.78, *p* = 0.461) did not differ significantly between groups. On day 2, the experiment was terminated prematurely for eight participants at their own request or due to technical difficulties (each 2 from the UE and VEI group and 4 from the VEI + R group) leaving a sample of *N* = 112 for the second day of measurement, again without significant differences in gender (Χ² < 1) or age (*F* < 1) distribution. Analyzed samples can differ dependent on the outcome measures and are reported in the corresponding sections.

### Experimental design and procedure

The differential cue conditioning paradigm spanned two sessions on consecutive days, approximately 24 hours apart (tolerance range ± 2 hours). On day 1, participants took part in a fear acquisition and extinction training, and on day 2, we tested for spontaneous recovery and reinstatement of fear as described below (see also Fig. 1). Two faces with neutral expressions from the Radbound Faces Database (RaFD, Nos. Rafd090_71 and Rafd090_26)^38^ served as CSs and were presented for seven seconds. Two different orders mirrored each other with regard to CS+ and CS− presentations to account for initial sensitization and subsequent habituation effects and were balanced between participants. CS+ and CS− presentations were pseudo-randomized with no more than two consecutive presentations of the same condition. Assignment of each the faces as threat (CS+) or safety (CS-) signal was also balanced between participants. The US was a 10 ms train of 500 Hz single electrical pulses (1 ms; S48K; Grass Instruments, West Warwick, RI), applied to the participants’ left ankle, adjusted for each participant individually to a level that was “highly unpleasant, but not painful” and presented 6.95 sec after onset of the CS+. The mean physical intensity of the US was 21.78 mA (*SD* = 20.80) and did not differ significantly between the three groups (*F*(2,117) = 1.244, *p* = 0.292). The acoustic startle probe was a 95 dB(A) white noise, presented 5.5 or 6.5 sec after cue onset and 11, 12 or 13 sec after cue offset during inter-trial intervals (ITIs) for 50 ms. The duration of ITIs, during which a fixation cross was presented, varied between 11 and 18 sec. The presentation of visual stimuli was controlled by Presentation (version 17), presentations of startle probes and USs by VPM^39^.

#### Day 1

Upon arrival in the laboratory, participants were informed about materials and procedures and signed a corresponding consent form. Afterwards the sensors for physiological data recording were attached and an electrocardiogram (ECG) was recorded for six minutes to determine resting heart rate variability (HRV; findings on the association between resting HRV and instructed extinction have been reported elsewhere^37^.). Then, four startle probes were presented without visual foreground stimuli to account for startle habituation effects (habituation 1), followed by a pre-conditioning phase in which startle probes were administered during four presentations of each of the CS and additionally four times during the ITIs. Afterwards, participants rated the valence and arousal of each of the CS as well as the startle probe to determine a baseline liking of the CS. Then the US electrode was attached and the US intensity was individually adjusted. Participants were informed that all stimuli (visual, auditory, electrotactile) would be presented in the following experimental phase and that they have no special task other than keeping their eyes open and focusing on the screen. The acquisition training was preceded by four startle probes presented without visual foreground stimuli to account for sensitization effects due to the US work-up procedure (habituation 2). During the acquisition training 9 out of 12 CS+ trials were reinforced by US presentations, while 12 CS- trials never included US presentations. Then the examiner entered the room, valence and arousal ratings were obtained for each of the CSs as well as for the startle probe and the US and participants were instructed according to their group assignment. Participants of the UE group were told that the experiment will continue after a short break and both participants of the VIE and the VIE-R group were instructed that no further US will be applied. In the VIE-R group the US electrode was additionally removed which was accompanied by an explicit statement that the US electrode is now removed. During the subsequent extinction phase, CS+ and CS− were presented 12 times each with no further US presentations. Startle probes were administered during all CS+ and CS− presentations as well as during a corresponding number of ITIs, i.e., each 12 during the acquisition and the extinction training. Afterwards, valence and arousal ratings were obtained again, participants were interviewed to determine their awareness of CS-US contingencies. Overall, 11.67% of the participants were unaware of CS-US contingencies and the proportion of unaware participants did not differ between extinction groups (Χ²(2) = 2.102, *exact p* = 0.455).

#### Day 2

The second session began 24 ± 2 hours after the first with another six minute resting period for ECG recording after the sensors for physiological data recording were reattached. After four presentations of startle probes without visual foreground stimuli to account for startle habituation effects, spontaneous recovery of the CR while the US electrode was not attached (re-exposure 1), participants were re-exposed to each four CS+ and CS− presentations. Startle probes were presented in all CS and in four ITI trials. Then, valence and arousal ratings were obtained, the US electrode was reattached and participants were informed that all stimuli experienced the day before could be presented in the following experimental phases. To test for spontaneous recovery of the CR while the US electrode was attached (re-exposure 2), participants were again re-exposed to each four CS+ and CS− presentations and startle probes were presented in all CS and in four ITI trials. Afterwards one unsignaled US presentation during an ITI was used to test the reinstatement of fear by re-exposure to the aversive event. Whether that ITI was preceded and followed by CS+ or CS- trials was balanced between participants. Startle probes were presented in all 12 CS+ and 12 CS- trials that followed and additionally 12 times during the ITIs. No further US were applied during the final experimental phase. Then valence and arousal ratings were obtained again and participants completed a set of questionnaires including German versions of the State Trait Anxiety Inventory (STAI)^40^, the Childhood Trauma Questionnaire (CTQ)^41^, the List of Threatening Experiences (LTE)^42^ and the Resilience Scale (RS-11)^43^. Trait anxiety (STAI: *F*(2,114) = 0.22, *p* = 0.805), childhood adversity (CTQ: *F*(2,115) = 1.36, *p* = 0.261), number of threatening experiences in the last twelve months (LTE: *F*(2,114) = 0.00, *p* = 0.999) and resilience subsumed as coping with change or misfortune (RS-11: *F*(2,111) = 0.51, *p* = 0.603) did not differ significantly between groups.

### Assessment and processing of outcome measures

#### Startle blink responses

Electromyographic (EMG) activity was recorded over the left orbicularis oculi muscle to measure the eyeblink component of the startle response using two Ag/AgCl miniature surface electrodes (4 mm diameter; Grass Products) filled with electrolyte (Marquette Hellige). A Coulbourn S75-01 bioamplifier served to amplify the raw EMG with a 30 Hz high-pass filter and a 400 Hz Kemo-VBF8-03 low-pass filter. Digital sampling was set to 1000 Hz from 100 ms prior to until 400 ms after onset of the startle probe. The EMG signal was digitally filtered offline through a 60 Hz high-pass filter and was rectified and integrated (time constant: 10 ms).

Startle blink responses were defined when starting 20–120 ms after probe onset and peaking within 150 ms using a custom made computer algorithm^44^ that automatically identified latency of blink onset and peak amplitude in microvolts. All trials were visually inspected and trials with no detectable blinks were scored as zero responses, trials with excessive baseline activity or recording artefacts as missing (0.03% of trials in the analyzed sample). To reduce the influence of individual differences unrelated to the research question, blink magnitudes were standardized for each participant using a z-score transformation with all blinks from both days as a reference distribution^45^. The standardized responses of each participant were then converted to T scores [50 + (*z* × 10)].

Due to technical difficulties during EMG measurement, FPS data from nine (day 1) resp. 11 (day 2) participants were excluded from analysis. Another two (day 1) resp. one (day 2) participant(s) were excluded from analysis because they failed to reach a startle probability (1 - (N zero responses/(N probes - N missings))^42^ of >50% (analyzed sample day 1 *N* = 109, analyzed sample day 2 *N* = 100).

#### Skin conductance responses

Electrodermal activity (EDA) was recorded from the hypothenar eminence of the palmar surface of the participant’s right hand. A Coulbourn S71-22 skin conductance coupler provided a constant 0.5 V across two Ag/AgCl standard electrodes (8 mm diameter; Gelimed) filled with a 0.05 M sodium chloride electrolyte medium. The analog signal was sampled continuously at a rate of 10 Hz. SCRs were scored from trough-to-peak as the first increase starting between 0.9 and 4.0 s after cue onset (first interval response; minimal response criterion = 0.04 μS) and within the presentation of the CSs (7 s) to avoid confounding with SCRs to the USs, again using a custom made computer algorithm^44^.

Due to technical difficulties during EDA measurement, data from nine (day 1) resp. seven (day 2) participants were missing for SCR analysis. Another three participants were defined as SCR non-responder because they showed no increase in skin conductance in response to more than 50% of US presentations and excluded from all further analysis (analyzed sample day 1 *N* = 108, analyzed sample day 2 *N* = 102). SCRs were square-root-transformed to compensate for skewed distributions and range-corrected based on the maximum SCR (including both CRs and URs) prior to statistical analyses.

#### CS valence ratings

Valence and arousal ratings were obtained for CS, startle probe and US stimuli three times on day 1 (after the pre-conditioning phase, the acquisition training and the extinction training respectively) and two times on day two (after the first re-exposure the CSs and after the final experimental phase which included the second re-exposure the CSs with the US electrode attached, the unsignaled US presentation and 12 further presentation of both the CS+ and the CS-). Ratings were acquired from 1 (very pleasant/calm) to 9 (very unpleasant/arousing) using the Self-Assessment Manikin Scale (SAM)^46^. For the purpose of this manuscript, we focus on CS valence ratings as a measure of evaluative learning.

### Statistical analysis

All measures were checked for baseline group differences which were then entered as covariates in the corresponding analyses if appropriate. Responses during pre-conditioning served as baseline for the analyses of the acquisition and extinction phases on day 1, responses during the last two trials of extinction training as baseline for the analyses of the re-exposure phases on day 2.

Startle blink magnitudes were analyzed using linear mixed models as implemented in IBM SPSS Statistics (Version 22) with the repeated covariance structure set to AR1 (autocorrelated) to account for expected timing-dependent differences in correlations between observations in this learning paradigm. Partial R² was calculated for significant effects as a measure of effect size for fixed effects^47^. In case of significant main effects and more than two factor levels, Sidak-corrected pairwise comparisons are reported along with 95% confidence intervals. In case of significant interactions, follow-up linear mixed models were used to explore their nature. In case of non-significant effects, Bayes factors were calculated as implemented in Version 25 of IBM SPSS Statistics to determine the likelihood of the data under the null hypothesis of no effect and Bayes factors lower than 1/3 were considered as support for the null model^48^.

To test for baseline differences between groups, startle blink magnitudes during pre-conditioning were tested for the fixed effects trial type (CS+ vs. CS- vs. ITI) and instruction group (VIE vs. VIE+ R vs. UE). To account for learning affects during acquisition and extinction training, trial number (trials 1 to 12) introduced as fixed effects as well as trial type and instruction group into the respective models. To test for immediate extinction effects, a separate analysis was run testing the fixed effects trial type and instruction group on the first extinction trial only. The model for return of fear on day 2 included the fixed effects trial type, US electrode reattachment (pre vs. post) and instruction group. Reinstatement effects were tested with a model that included the fixed effects trial type, reinstatement US (pre-reinstatement trial vs. post-reinstatement trials) and instruction group. Since only one trial per condition was included in the reinstatement model^33^, the repeated covariance structure was set to compound symmetry here.

SCRs were analyzed as described for startle blink magnitudes except that the three-level factor trial type was replaced by the two-level factor CS type (CS+ vs. CS-) since no SCRs were obtained during ITIs. CS valence ratings were analyzed in three linear mixed models with the repeated covariance structure set to compound symmetry: The first one to check for baseline differences during pre-conditioning with CS type and instruction group introduced as fixed effects, the second model was used to test learning effects on day 1 with the fixed effects CS type, time of query (assessment after acquisition training vs. after extinction training) and instruction group and the third model to test for return of fear on day 2 with the fixed effects CS type, time of query (assessment after re-exposure 1 vs. after completion of the experiment) and instruction group.

## Data Availability

Data will be made available by the corresponding author upon reasonable request.
